# Insights into Xylan Degradation and Haloalkaline Adaptation through Whole-Genome Analysis of *Alkalitalea saponilacus*, an Anaerobic Haloalkaliphilic Bacterium Capable of Secreting Novel Halostable Xylanase

**DOI:** 10.3390/genes10010001

**Published:** 2018-12-20

**Authors:** Ziya Liao, Mark Holtzapple, Yanchun Yan, Haisheng Wang, Jun Li, Baisuo Zhao

**Affiliations:** 1 Graduate School, Chinese Academy of Agricultural Sciences, Beijing 100081, China; ziyaliao@163.com (Z.L.); yanyanchun@caas.cn (Y.Y.); wanghaisheng@caas.cn (H.W.); 2 Department of Chemical Engineering, Texas A&M University, College Station, TX 77843, USA; m-holtzapple@mail.che.tamu.edu; 3 Institute of Agricultural Resources and Regional Planning, Chinese Academy of Agricultural Sciences, Beijing 100081, China; junli01@caas.cn

**Keywords:** Genome Sequencing, Haloalkaliphile, Xylanase, *Alkalitalea saponilacus*

## Abstract

The obligately anaerobic haloalkaliphilic bacterium *Alkalitalea saponilacus* can use xylan as the sole carbon source and produce propionate as the main fermentation product. Using mixed carbon sources of 0.4% (*w*/*v*) sucrose and 0.1% (*w*/*v*) birch xylan, xylanase production from *A. saponilacus* was 3.2-fold greater than that of individual carbon sources of 0.5% (*w*/*v*) sucrose or 0.5% (*w*/*v*) birch xylan. The xylanse is halostable and exhibits optimal activity over a broad salt concentration (2–6% NaCl). Its activity increased approximately 1.16-fold by adding 0.2% (*v*/*v*) Tween 20. To understand the potential genetic mechanisms of xylan degradation and molecular adaptation to saline-alkali extremes, the complete genome sequence of *A. saponilacus* was performed with the pacBio single-molecule real-time (SMRT) and Illumina Misseq platforms. The genome contained one chromosome with a total size of 4,775,573 bps, and a G+C genomic content of 39.27%. Ten genes relating to the pathway for complete xylan degradation were systematically identified. Furthermore, various genes were predicted to be involved in isosmotic cytoplasm via the “compatible-solutes strategy” and cytoplasmic pH homeostasis though the “influx of hydrogen ions”. The halostable xylanase from *A. saponilacus* and its genomic sequence information provide some insight for potential applications in industry under double extreme conditions.

## 1. Introduction

Haloalkaliphiles are extremophilic microorganisms that grow optimally above 0.5 mol·L^–1^ salinity (NaCl) and above pH 9.0 (sodium carbonate/sodium bicarbonate) [1,2]. They are naturally found in saline-alkaline environments such as soda lakes and soda deserts in various dry steppes and semi-desert areas around the world. They also are found in human industrial processes, such as those involving mineral ore, petroleum refining, pulp and paper, textile preparation, leather tanneries, food and potato processing units, lime kilns, and detergent manufacture, all of which generate effluents containing NaOH, Ca(OH)_2_, etc. [1,3,4]. Over the last three decades, there has been increased interest in exploring haloalkaliphiles as a precious resource that produces stable unique exo-enzymes and organic compounds with potential applications in various industrial processes [1,5,6]. However, to date, our knowledge of anaerobic haloalkaliphiles associated with exploitable enzymology and genetic adaptations is still limited. The genome sequences of haloalkaliphiles may enable many new and potentially transformative biotechnological efforts by providing genetic information to meet rapidly growing industrial demands.

The obligately anaerobic haloalkaliphilic xylanolytic bacterium *Alkalitalea saponilacus* SC/BZ-SP2^T^, grows optimally at 0.44–0.69 mol·L^–1^ Na^+^ (equivalent to 2.6–4.0% NaCl) and pH 9.7. It was retrieved from a meromictic soda lake [7]. This microorganism is classified as a species in genus *Alkalitalea*, family *Marinilabiliaceae*, class *Bacteroidia*, order *Bacteroidetes*. The haloalkaliphile *A. saponilacus* is the first identified anaerobic bacterium that uses xylan as the sole carbon and energy source, and simultaneously produces propionic acid as the major product. If this xylanase is excreted into highly saline-alkaline surroundings and is easily recovered, perhaps it can be applied in industry, such as the biobleaching of wood pulp. This may be the first report about the complete genome sequence of *A. saponilacus*, which could be used by industry in the future.

## 2. Materials and Methods

### 2.1. Concentration and Characterization of the Xylanase

To achieve more in-depth understanding of the xylanase characteristics, *A. saponilacus* SC/BZ-SP2^T^ was optimally grown using birch xylan, sucrose, maltose, glucose, and cellobiose as sole carbon sources as described previously by Zhao and Chen [7]. The bacterial culture (20 mL) was mildly ultra-sonicated in an ice bath for 10 min with 3-s intervals while emitting 200 W (Branson digital sonifier 250, Branson Ultrasonics, Danbury, CT, USA). For crude concentration, the xylanse suspended in culture media was precipitated using a 40% saturated solution of ammonium sulfate and centrifuged for 20 min at 9425× *g* (i.e., 10,000 rpm) at 4 °C.

Xylanase activity was measured using xylose as the standard with the modified 3, 5-dinitrosalicylic acid colorimetric method (DNS method) [8]. One unit (1 U) of purified xylanase activity was defined as the amount of enzyme that released 1 μmol of xylose equivalent per min under the assay conditions. The relative xylanase activity is defined as the percentage of the maximum xylanase activity measured at various experimental conditions. The conditions for optimal xylanase activity were assayed as follows. (1) NaCl concentrations (0–22%, *w*/*v*, at intervals of 2%) at pH 7.0 and at 55 °C; (2) temperatures (30–90 °C, at intervals of 5 °C) with 4% NaCl and pH 7.0; and at pH (4.0–10.5 with intervals of 0.5 pH units) using sodium citrate buffer (pH 4.0–6.0), sodium phosphate buffer (pH 6.0–8.0), and glycine–NaOH buffer (pH 8.0–10.5) at 4% NaCl and at 55 °C. In addition, the effects of surfactants (0.2%, *v*/*v*) and various metals (5 mM) on xylanase activity were tested at optimal conditions (i.e., 4% NaCl, pH 7.0, and 55 °C).

### 2.2. Genome Sequencing, Annotation and Analysis Pipelines

To obtain detailed genetic information of xylan degradation and saline-alkali tolerance, the whole genome of *A. saponilacus* was completely sequenced. Genomic DNA (5 μg) was extracted using Itop^TM^ microbial DNA isolation kit (Itop, Beijing, China) according to the manufacturer’s instructions (Beijing, China). After clone library construction, with a mean size of 8–10 kb using g-Tubes, genome sequencing was performed on a Pacific Biosciences RS II sequencer (Pacific Biosciences, Melon Park, CA, USA) using the SMRTbell temperate prep kit version 1.0 (Pacific Biosciences, Menlo Park, CA, USA) and loaded onto a single-molecule real-time (SMRT) cell (Pacific Biosciences, Menlo Park, CA, USA). All cleaned reads were de novo assembled using Hierarchical Genome Assembly Process (HGAP 2.0) [9], resulting in a single contiguous sequence. Briefly, single reads were mapped to seed reads, overlapping consensus sequences were created by a Celera assembler 8.0 [10,11], and the remaining indel and base substitution errors were removed. This method can produce highly accurate and complete de novo assemblies for small prokaryotic genomes [12]. Additionally, libraries were constructed using the TruSeq Nano DNA library preparation kit (Illumina, San Diego, CA, USA) and used to generate 150-bp paired-end reads via the Illumina HiSeq 2000 platform (Illumina, San Diego, CA, USA). Remapping quality-filtered Illumina reads was performed by onto the assembly using BWA [13]. The alignment was passed to Pilon [14] to correct for indels and single nucleotide polymorphisms (SNPs). The average read length of the pacBio raw data was ~6.6 kb, with maximum read length of about 41,148 bases (coverage, ~498) and Illumina paired-end sequencing generated 1.1 million with 2 × 150-bp reads (coverage, ~150). The whole complete genome sequence of *A. saponilacus* SC/BZ-SP2^T^ has been deposited at DDBJ/ENA/GenBank under the accession number CP021904.

Glimmer 3.02 was used for gene prediction, gene number, gene total length, and so on [15]. Automated gene annotation was obtained though NCBI Prokaryotic Genome Annotation Pipeline (PGAP) [16]. Then, the genome files in GenBank format (gb file) were uploaded to the Integrated Microbial Genomes Expert Review (IMG/ER) tool [17] for functional annotation, followed the registration of IMG Analysis Project ID (Ga0265418), and Submission ID (184155) in the Genomes OnLine Database (Gold) [18]. Clusters of orthologous groups of proteins (COG) analyses were undertaken using COG functions and abundance profile analysis within IMG/ER [17]. Kyoto encyclopedia of genes and genomes (KEGG) pathway was analyzed using the online tool [19,20].

## 3. Results and Discussion

### 3.1. Characteristics of Alkalitalea saponilacus Xylanase

**Figure 1:**
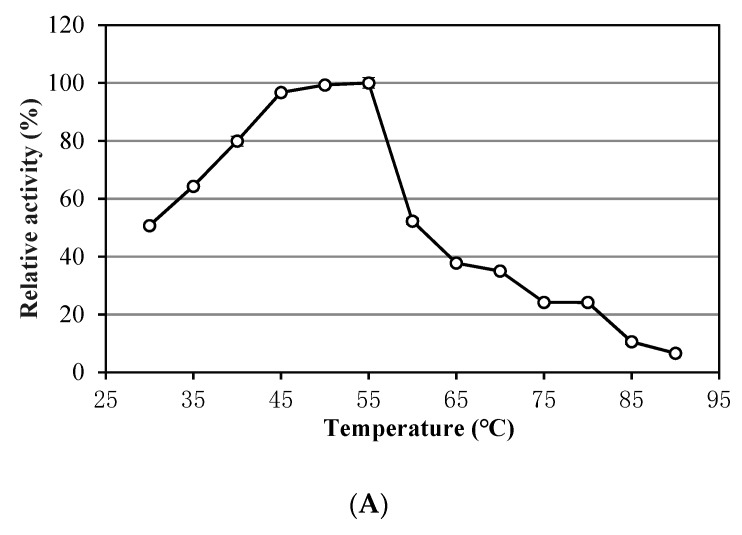

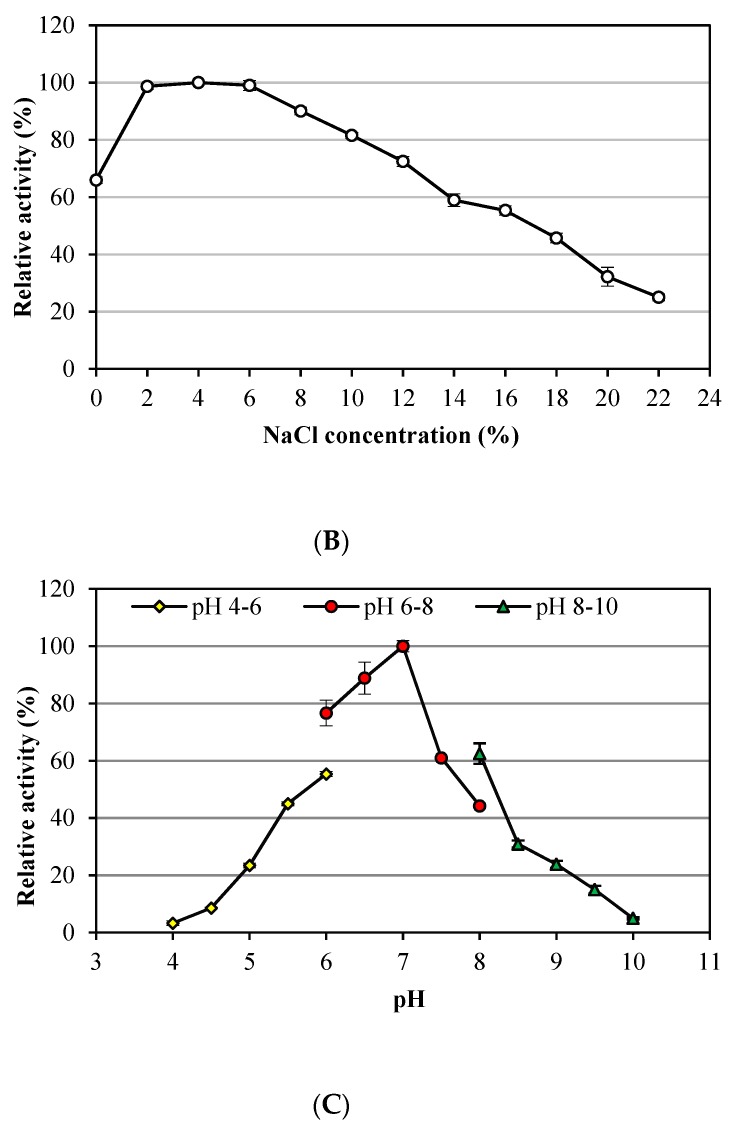
Characteristics of xylanase produced by *Alkalitalea saponilacus.* (**A**) Influence of temperature on xylanase activity, (**B**) Influence of different NaCl concentration on xylanase activity, (**C**) Influence of pH on xylanase activity. Buffer solutions are citric acid-sodium citrate buffer solution (pH 4–6), sodium hydrogen phosphate-sodium dihydrogen phosphate buffer solution (pH 6–8), glycine-sodium hydroxide buffer solution (pH 8–10), respectively. The error bars indicate standard deviation (SD) of triplicate determination.

Noticeably, *A. saponilacus* can use insoluble unsubstituted xylan as the sole substrate, indicating it can secrete extracellular xylanase into the surroundings [21]. This microorganism produces xylanase when using birch xylan, sucrose, maltose, glucose, and cellobiose as carbon sources [7]. Xylanase production with a mixture of 0.4% (*w*/*v*) sucrose and 0.1% (*w*/*v*) birch xylan substrates was 3.2 greater times than individual carbon sources of 0.5% (*w*/*v*) sucrose or 0.5% (*w*/*v*) birch xylan. This may reduce production costs of industrial xylanase because sucrose is widely distributed and less expensive [22]. Figure 1 shows the *relative* xylanase activity with respect to temperature of 30–90 °C (a broad optimum temperature of 45–55 °C), NaCl concentration of 0–22% (*w*/*v*) (a wide optimum range of 2–6%), and pH of 4.0–10 (optimum pH 7.0). This xylanase tolerates high temperature, acidic and alkali conditions with its unique halophilic characteristic. Unfortunately, it is not alkaliphilic, which was unexpected based on previous descriptions. However, it is not uncommon because xylanase produced from alkaliphilic *Bacillus* sp. Strain K-1 also has an optimal activity at acidic pH 5.5 [21]. Xylanase activity tolerates surfactant (0.2% *v*/*v*) such as Tween 20 and Triton X-100; its activity increased by 1.16 times with addition of Tween 20. In addition, activity is inhibited by 5-mM metal ions of Cu^2+^, Fe^3+^, Ni^2+^, Al^3+^, Mn^2+^, Co^2+^, Zn^2+^, and Ca^2+^ with no influence from Mg^2+^.

### 3.2. Genome Features of Alkalitalea saponilacus

The complete genome of *A. saponilacus* contained only one chromosome with 4,775,573 bp. The genomic G+C content was 39.27% (Table S1 and Figure S1). Among the 3688 genes predicted, 3626 protein-coding genes (CDS) were predicted that accounted for 98.32% of the whole genome, and 74.02% of which (2684 CDS) were functionally annotated. Also, 12 rRNA genes (four 5S RNAs, four 16S RNA, and four 23S RNA), 48 tRNA genes, and two noncoding RNAs (ncRNAs) were identified.

Of the total 3626 predicted protein-coding genes, 2225 genes (60.33% of the total) were assigned to Clusters of Orthologous Groups (COGs) of proteins (Table S2) and distributed into 23 different categories. The cluster for “cell wall/membrane/envelope biogenesis (COG M)” (200, accounting for 8.99% COGs genes) was the largest group, followed by the classification of “carbohydrate transport and metabolism (COG G)” (181, 8.13%), “translation, ribosomal structure and biogenesis (COG J)” (178, 8.00%) and “amino acid transport and metabolism (COG E)” (159, 7.15%). The categories of “extracellular structures (COG W)” (5, 0.22%) and “cytoskeleton (COG Z)” (1, 0.04%) were the smallest groups.

### 3.3. The Identified Xylan-Degrading Related Enzymes in Alkalitalea saponilacus

**Figure 2:**
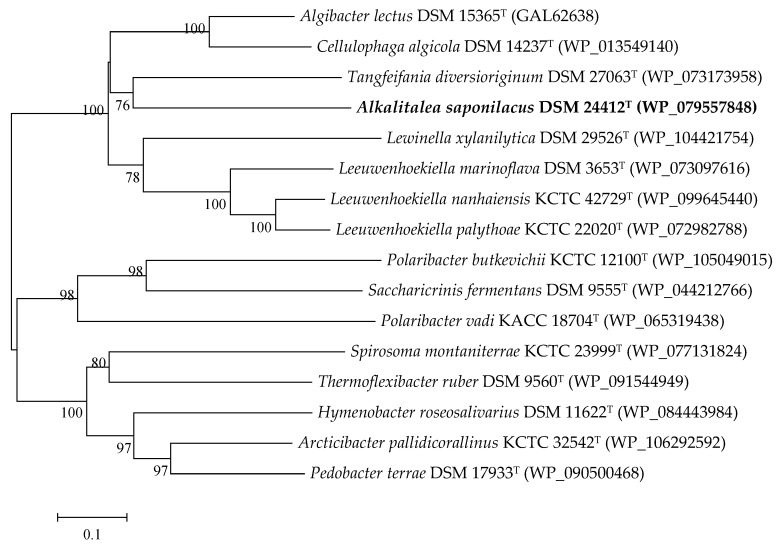
Neighbor-joining phylogenetic tree based on 16 xylanases sequences by using MEGA (Version 6). Numbers on nodes correspond to percentage bootstrap values for 1000 replicates.

To completely degrade xylans with various substitutions, three major hydrolytic enzymes (endo-*β*-1,4-xylanases, *β*-xylosidases, and *α*-glucuronidase) and several accessory enzymes (e.g., *α*-L-arabinofuranosidases, *α*-glucosidases uronidases, and acetyl and feruloyl esterases) might be necessary [23,24]. Genome analysis of *A. saponilacus* revealed one gene of endo-*β*-1,4-xylanase (XynA), six genes of *β*-xylosidase (GH43), one gene of *α*-glucuronidase, and four genes of *α*-L-arabinofuranosidase (Table 1). Absent were genes encoding acetylxylan esterase, ferulic acid esterase, and *p*-coumaric acid esterase. XynA, which belongs to glycoside hydrolase family 10 (GH10), is a crucial enzyme during xylan degradation and is responsible for fracturing the heteroxylan backbone [25]. XynA of *A. saponilacus* shared the highest sequence identity (55.2%) with the corresponding protein (RefSeq: WP_073173958) of *Tangfeifania diversioriginum* DSM 27063^T^, and was 52.4% (WP_074935698) and 52.3% (WP_013549140) identical to that of *Algibacter lectus* DSM 15365^T^ and *Cellulophaga algicola* DSM 14237^T^, respectively. Furthermore, a phylogenetic tree generated from amino acid sequences of 16 xylanase were constructed with neighbor-joining algorithms [26] in MEGA version 7 [27] (Figure 2). The phylogenetic tree showed XynA of *A. saponilacus* formed a single cluster with that of *T. diversioriginum* DSM 27063^T^ (bootstrap support 76%), and separate branch from those of both *Algibacter lectus* DSM 15365^T^ and *Cellulophaga algicola* DSM 14237^T^, suggesting XynA of *A. saponilacus* may possess distinct characteristics. 

### 3.4. The Predicted Xylan Degradation Pathways in Alkalitalea saponilacus 

**Figure 3:**
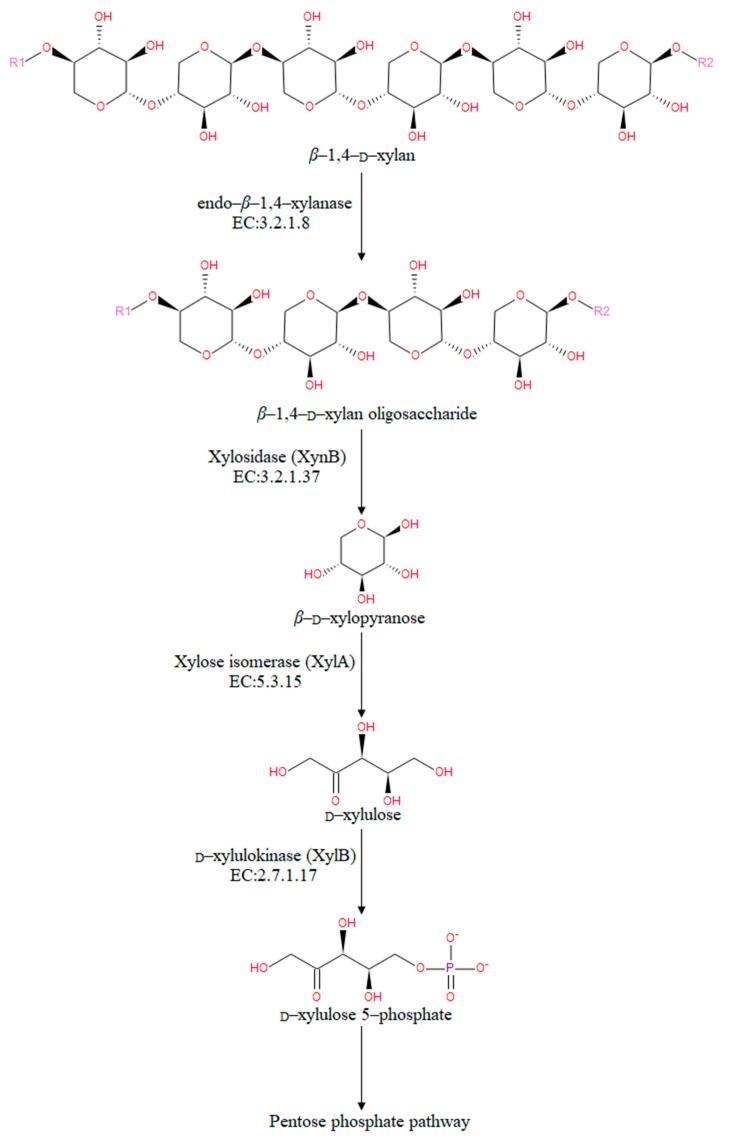
Enzymatic steps of xylan degradation pathways in *A. saponilacus.*

To identify xylan degradation pathways in *A. saponilacus*, the 3688 annotated gene sequences to reference canonical pathways in KEGG were mapped and a total of 163 KEGG pathways were obtained. Based on KEGG annotation for genes of potential xylan-degrading enzymes (e.g., endo-*β*-1,4-xylanase and xylosidase), other potential enzymes involved in xylan degradation (e.g., xylose isomerase and D-xylulokinase) were also identified *A. saponilacus* (Figure 3). Using the genome sequence of *A. saponilacus*, these enzymes can be molecularly cloned to study their inherent characteristics and be modified through genetic engineering technology to be applied into industry.

Under the action of the above enzymes, XynA may possess a putative signal peptide containing 19 amino acids that can guide this enzyme to be secreted outside of cell. *Thermotoga neapolitana*, which was the highest identity sequence, has a similar extracellular enzyme that is secreted into the medium to degrade the macromolecular xylan to xylooligosaccharides, which are transported back into the cell through the specific oligosaccharide transport systems for further assimilation [25]. With the action of XynA, the glycosidic linkage (*β*-1,4) of xylosides is broken first, and *β*-1,4-_D_-xylan oligosaccharides form. Next, xylosidase removes xylose residues from the nonreducing end of *β*-1,4-_D_-xylan oligosaccharides, leading to the release of D-xylose, which then converted into D-xylulose using xylose isomerase (XylA). After that, the phosphate and energy from hydrolysis of adenosine triphosphate (ATP) facilitates the conversion of D-xylulose to D-xylulose 5-phosphate, which then is used by the pentose phosphate pathway (PPP). Finally, the D-xylulose 5-phosphate may be transformed into propionic acid using other metabolic pathways [28]. Based on gene annotation, the above pathways are proposed as the way that xylan is metabolized by *A. saponilacus*.

### 3.5. The Genes Involved in Adaptation to Saline-Alkaline Conditions in Alkalitalea saponilacus

Genome sequence analysis also showed that there are many genes that encode putative proteins potentially associated with the adaptation of *A. saponilacus* to saline-alkaline conditions (Table 2). The presence of *glnA* gene encoding for L-glutamine synthase, one gene coding for choline/glycine/proline betaine transporter (BCCT family), and four genes for Na^+^/solute symporter (SSS family) indicates that *A. saponilacus* maintains osmotic equilibrium across membranes using the “compatible-solutes strategy” when exposed to high salinity [1,29,30,31,32]. Additionally, four genes affiliated with the Trk family (two-TrkA-type and two-TrkH-type) responsible for K^+^ uptake systems were found, which implies that that *A. saponilacus* might achieve an isosmotic cytoplasm using K^+^ to cope rapidly with an osmotic shock [33]. To survive in highly alkaline conditions, *A. saponilacus* has developed genetic adaptations for pH homeostasis via the “influx of hydrogen ions” [34,35,36]; it harbors seven genes of multisubunit Na^+^/H^+^ antiporter, four genes of monovalent cation/H^+^ antiporter (CPA family, two-CPA1-type and two-CPA2-type), and one gene of Na^+^/H^+^ antiporter (NhaC family). Eight genes encoding for F_0_F_1_-ATP synthase and six genes coding for H^+^-transporting ATPase (V/A-type) allow *A. saponilacus* to maintain a constant hyper pH cytoplasm using proton gradients [37]. As described above, various predicted genes in *A. saponilacus* offer valuable insights to reveal the adaptive mechanisms of this haloalkaliphile.

## 4. Conclusions

This strain of *A. saponilacus* can grow anaerobically using xylan as the sole carbon source at hypersaline and extremely alkaline conditions. The xylanase activity with the combined substrates of 0.4% sucrose + 0.1% birch xylan substrates was significantly higher than with individual substrates of sucrose or birch xylan. Optimum xylanase activity was obtained at 2–6% NaCl, pH7.0, and 45–55 C. Xylanase activity increased by 1.16 times with addition of Tween 20 whereas it was inhibited by 5-mM Cu^2+^, Fe^3+^, Ni^2+^, Al^3+^, Mn^2+^, Co^2+^, Zn^2+^, and Ca^2+^. The genome sequence of *A. saponilacus* has revealed much about the many adaptations of this haloalkaliphile, which allows it to degrade xylan and live in extreme environments. The metabolic enzymes related to xylan degradation, particularly endo-*β*-1,4-xylanase (XynA), is a new resource of enormous potential value with halophilic characteristics. By synthesizing and transporting compatible solutes, *A. saponilacus* can maintain osmotic equilibrium and survive in hypersaline environments. The chromosome has a wealth of genes that allow Na^+^, H^+^, and K^+^ to be imported and exported, which achieves an isosmotic cytoplasm that adapts to hypersaline environments. 

## Figures and Tables

**Table 1:** The identified xylan-degradation-related enzymes. All locus tag numbers are predicted and indicated by IMG/ER. GH: glycoside hydrolase.

Locus Tag	Product Name	GH
CDL62_17705	Endo-*β-*1,4-xylanase (XynA)	GH10
CDL62_00085	*β*-xylosidase	GH43
CDL62_06240	*β*-xylosidase	GH43
CDL62_06275	*β*-xylosidase	GH43
CDL62_06380	*β*-xylosidase	GH43
CDL62_15875	*β*-xylosidase	GH43
CDL62_02285	*β*-xylosidase	GH43
CDL62_00095	*α*-glucuronidase	GH67
CDL62_00195	*α*-L-arabinofuranosidase	GH43
CDL62_00495	*α*-L-arabinofuranosidase	GH43
CDL62_12950	*α*-L-arabinofuranosidase	GH43
CDL62_00395	*α*-L-arabinofuranosidase	GH51

**Table 2:** Genes in *A. saponilacus* involved in adaptation to saline-alkaline environments.

Product Name	Locus Tag
L-glutamine synthesis	
L-glutamine synthetase, GlnA	CDL62_11360
Choline/glycine/proline betaine transporter (BCCT family)	
Choline/glycine/proline betaine transport protein	CDL62_17705
Na^+^/solute symporter	
Na^+^/solute symporter (SSS family)	CDL62_09935
Na^+^/solute symporter (SSS family)	CDL62_06475
Na^+^/solute symporter (SSS family)	CDL62_14105
Na^+^/solute symporter (SSS family)	CDL62_11075
K^+^ transport systems, potassium uptake protein (Trk family)	
Trk system potassium uptake protein, TrkA	CDL62_03510
Trk system potassium uptake protein, TrkH	CDL62_03515
Trk system potassium uptake protein, TrkA	CDL62_03555
Trk system potassium uptake protein, TrkH	CDL62_12070
Na^+^/H^+^ antiporter (NhaC family)	
H^+^/Na^+^ antiporter (NhaC family)	CDL62_06020
Multisubunit Na^+^/H^+^ antiporter	
Multisubunit Na^+^/H^+^ antiporter, MrpA subunit	CDL62_14320
Multisubunit Na^+^/H^+^ antiporter, MrpB subunit	CDL62_14325
Multisubunit Na^+^/H^+^ antiporter, MrpC subunit	CDL62_14330
Multisubunit Na^+^/H^+^ antiporter, MrpD subunit	CDL62_14335
Multisubunit Na^+^/H^+^ antiporter, MnhE subunit	CDL62_14340
Multisubunit Na^+^/H^+^ antiporter, MnhF subunit	CDL62_14345
Multisubunit Na^+^/H^+^ antiporter, MrpG subunit	CDL62_14350
Monovalent Cation/H^+^ antiporter (CPA family)	
K^+^/H^+^ antiporter (CPA1 family)	CDL62_09425
K^+^/H^+^ antiporter (CPA1 family)	CDL62_00125
Na^+^/H^+^ antiporter (CPA2 family)	CDL62_00920
Na^+^/H^+^ antiporter (CPA2 family)	CDL62_05390
F_0_F_1_-ATP synthase	
ATP synthase F_1_ subcomplex gamma subunit, AtpG	CDL62_07555
ATP synthase F_1_ subcomplex alpha subunit, AtpA	CDL62_07560
ATP synthase F_1_ subcomplex delta subunit, AtpH	CDL62_07565
ATP synthase F_0_ subcomplex B subunit, AtpF	CDL62_07570
ATP synthase F_0_ subcomplex C subunit, AtpE	CDL62_07575
ATP synthase F_0_ subcomplex A subunit, AtpB	CDL62_07580
ATP synthase F_1_ subcomplex epsilon subunit, AtpC	CDL62_07660
ATP synthase F_1_ subcomplex beta subunit, AtpD	CDL62_07665
**H^+^-transporting two-sector ATPase (V-type ATP synthase)**	
V/A-type H^+^-transporting ATPase subunit E, AtpE	CDL62_11640
V/A-type H^+^-transporting ATPase subunit A, AtpA	CDL62_11650
V/A-type H^+^-transporting ATPase subunit B, AtpB	CDL62_11655
V/A-type H^+^-transporting ATPase subunit D, AtpD	CDL62_11660
V/A-type H^+^-transporting ATPase subunit I, AtpI	CDL62_11665
V/A-type H^+^-transporting ATPase subunit K, AtpK	CDL62_11670

## Supplementary Materials

The following are available online at http://www.mdpi.com/2073-4425/10/1/1/s1, Figure S1: Circular genome map of *Alkalitalea saponilacus*. The outermost ring of the circle indicates the size of genome, in which each scale represents 0.5 Mb. The second and third laps illustrate CDSs, colored by COG function classification. The second is forward strand, and the third is backward strand. The fourth circle denotes rRNA and tRNA. The fifth circle is GC content, the red part indicates that GC content is higher than average, whereas the blue is lower. The higher peak value heralds the greater difference in average GC content. The innermost circle states the GC skew (G-C/G+C). The plus-strand is more likely to transcribe CDS when the value is positive, yet minus strand tends to transcribe CDS when the value is negative; Table S1: Genome features of *Alkalitalea saponilacus* SC/BZ-SP2^T^; Table S2: Number of genes of *Alkalitalea saponilacus* associated with 23 general COG functional categories.
