# Supplementary material for: Insights into Xylan Degradation and Haloalkaline Adaptation through Whole-Genome Analysis of Alkalitalea saponilacus, an Anaerobic Haloalkaliphilic Bacterium Capable of Secreting Novel Halostable Xylanase
Source: Genes (Basel). 2018 Dec 20;10(1):1. doi: 10.3390/genes10010001 (PMC6357142; doi:10.3390/genes10010001)
Supplement: Supplementary file 1 [file genes-10-00001-s001.pdf]

Supplementary materials

Insights into Xylan Degradation and Haloalkaline Adaptation through Whole-Genome Analysis of *Alkalitalea saponilacus*, an Anaerobic Haloalkaliphilic Bacterium Capable of Secreting Novel Halostable Xylanase

Ziya Liao 1, Mark Holtzapple 2, Yanchun Yan 1, Haisheng Wang 1, Jun Li 3 and Baisuo Zhao 1,3,*

1 Graduate School, Chinese Academy of Agricultural Sciences, Beijing 100081, China; ziyaliao@163.com (Z.L.); yanyanchun@caas.cn (Y.Y.); wanghaisheng@caas.cn (H.W.)

2 Department of Chemical Engineering, Texas A&M University, College Station, TX 77843, USA; m-holtzapple@mail.che.tamu.edu

3 Institute of Agricultural Resources and Regional Planning, Chinese Academy of Agricultural Sciences, Beijing 100081, China; junli01@caas.cn

* Correspondence: bszhao@live.com

Received: 30 October 2018; Accepted: 13 December 2018; Published: date

Table S1. Genome features of *Alkalitalea saponilacus* SC/BZ-SP2^T.

Features	Statistics	
	**Value**	**% of total**
Length (bp)	4,775,573	100.00
G+C content (bp)	1,875,544	39.27
Coding region (bp)	4,118,672	86.24
Total genes	3,688	100.00
Protein coding genes	3,626	98.32
Pseudo genes	57	1.55
rRNA genes (5S, 16S, 2S)	4, 4, 4	0.33
tRNA genes	48	1.30
ncRNA genes	2	0.05
Genes with function prediction	2,684	72.78
Genes assigned to COGs	2225	60.33
Protein coding genes with enzymes	861	23.35

Table S2. Number of genes of *Alkalitalea saponilacus* associated with 23 general COG functional categories.

Code	Value	% of total	Description
Information storage and processing			
J	178	8.00	Translation, ribosomal structure and biogenesis
K	124	5.57	Transcription
L	90	4.04	Replication, recombination and repair
X	40	1.80	Mobilome: prophages, transposons
Cellular processes and signaling			
D	22	0.99	Cell cycle control, cell division, chromosome partitioning
M	200	8.99	Cell wall/membrane/envelope biogenesis
N	32	1.44	Cell motility
O	100	4.49	Posttranslational modification, protein turnover, chaperones
T	143	6.43	Signal transduction mechanisms
U	22	0.99	Intracellular trafficking, secretion, and vesicular transport
V	80	3.60	Defense mechanisms
W	5	0.22	Extracellular structures
Z	1	0.04	Cytoskeleton
Metabolism			
C	126	5.66	Energy production and conversion
E	159	7.15	Amino acid transport and metabolism
F	70	3.15	Nucleotide transport and metabolism
G	181	8.13	Carbohydrate transport and metabolism
H	133	5.98	Coenzyme transport and metabolism
I	81	3.64	Lipid transport and metabolism
P	123	5.53	Inorganic ion transport and metabolism
Q	28	1.26	Secondary metabolites biosynthesis, transport and catabolism
Poorly characterized			
R	200	8.99	General function prediction only
S	87	3.91	Function unknown

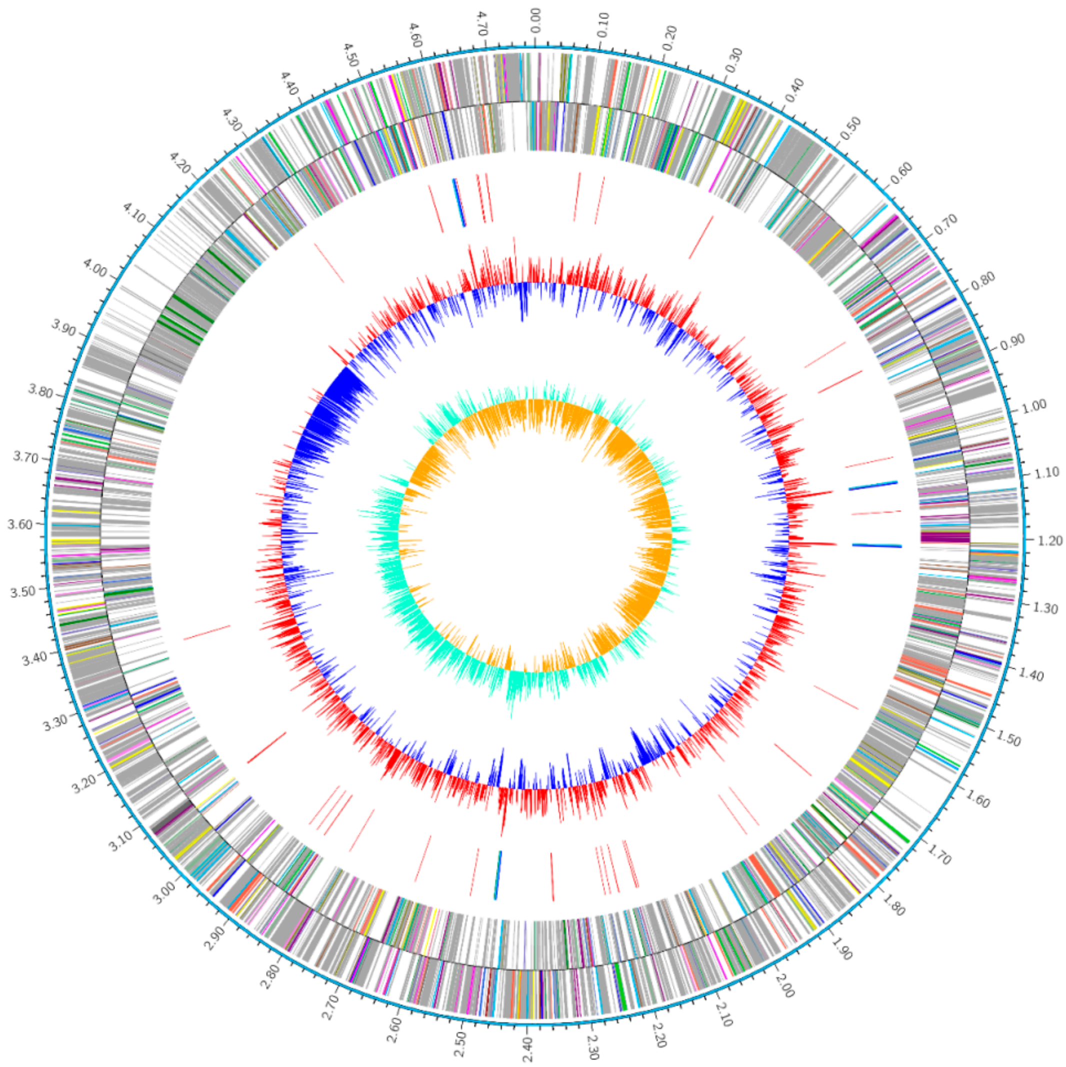

Figure S1. Circular genome map of *Alkalitalea saponilacus*. The outermost ring of the circle indicates the size of genome, in which each scale represents 0.5 Mb. The second and third laps illustrate CDSs, colored by COG function classification. The second is forward strand, and the third is backward strand. The fourth circle denotes rRNA and tRNA. The fifth circle is GC content, the red part indicates that GC content is higher than average, whereas the blue is lower. The higher peak value heralds the greater difference in average GC content. The innermost circle states the GC skew (G-C/G+C). The plus-strand is more likely to transcribe CDS when the value is positive, yet minus strand tends to transcribe CDS when the value is negative.
